# Prospective Randomized Trial to Assess Effects of Continuing Hormone Therapy on Cerebral Function in Postmenopausal Women at Risk for Dementia

**DOI:** 10.1371/journal.pone.0089095

**Published:** 2014-03-12

**Authors:** Natalie L. Rasgon, Cheri L. Geist, Heather A. Kenna, Tonita E. Wroolie, Katherine E. Williams, Daniel H. S. Silverman

**Affiliations:** 1 Stanford Center for Neuroscience in Women's Health, Department of Psychiatry and Behavioral Sciences, Stanford University School of Medicine, Stanford, California, United States of America; 2 UCLA David Geffen School of Medicine, Department of Molecular and Medical Pharmacology, Ahmanson Translational Imaging Division, University of California Los Angeles School of Medicine, Los Angeles, California, United States of America; University of Kansas Medical Center, United States of America

## Abstract

The objective of this study was to examine the effects of estrogen-based hormone therapy (HT) on regional cerebral metabolism in postmenopausal women (mean age = 58, SD = 5) at risk for development of dementia. The prospective clinical trial design included pre- and post-intervention neuroimaging of women randomized to continue (HT+) or discontinue (HT−) therapy following an average of 10 years of use. The primary outcome measure was change in brain metabolism during the subsequent two years, as assessed with fluorodeoxyglucose-18 positron emission tomography (FDG-PET). Longitudinal FDG-PET data were available for 45 study completers. Results showed that women randomized to continue HT experienced relative preservation of frontal and parietal cortical metabolism, compared with women randomized to discontinue HT. Women who discontinued 17-β estradiol (17βE)-based HT, as well as women who continued conjugated equine estrogen (CEE)-based HT, exhibited significant decline in metabolism of the precuneus/posterior cingulate cortical (PCC) area. Significant decline in PCC metabolism was additionally seen in women taking concurrent progestins (with either 17βE or CEE). Together, these findings suggest that among postmenopausal subjects at risk for developing dementia, regional cerebral cortical metabolism is relatively preserved for at least two years in women randomized to continue HT, compared with women randomized to discontinue HT. In addition, continuing unopposed 17βE therapy is associated specifically with preservation of metabolism in PCC, known to undergo the most significant decline in the earliest stages of Alzheimer's disease.

**Trial Registration:**

ClinicalTrials.gov NCT00097058

## Introduction

Estrogen has been shown to exert both neuroprotective and neuropathological effects in a variety of in vitro and in vivo models of brain disorders [1], [2]. Though controversial, studies on estrogen hormone therapy (HT) use in middle-aged and older postmenopausal women have suggested possible protection against aging-related cognitive decline, with the majority of previous studies reporting stabilization of cognitive and brain tissue integrity with HT [3], [4].

Notably, beneficial and detrimental findings in the literature to date suggest differential effects of HT by the respective type of estrogen formulation and the timing of initiation relative to the onset of menopause [5]. Many of the studies on HT that have found beneficial effects have included 17-β estradiol (17-βE)-based HT formulations [6]–[10], while the Women's Health Initiative Memory Study (WHIMS), which recruited women 65 and older, found an increased risk of dementia with conjugated equine estrogen (CEE) compared to placebo [11], [12]. Consequently, the WHIMS findings have resulted in the broad interpretation that HT fails to protect against dementia in postmenopausal women [11], though this conclusion conflicts with the majority of data showing beneficial effects of estrogen HT in the aging female brain [13]. The type of estrogen HT used by women has been proposed as a potential explanation for the discrepancy in the cumulative literature, as CEE and 17-βE-based preparations significantly differ in their biological action, especially in the brain [3], [4]. Further, there may also be a “window of opportunity” for beneficial effects of HT, as the observational studies based on typical prescription patterns (usually initiated during menopausal transition for treatment of vasomotor symptoms) of estrogen HT suggest a decreased risk for dementia with initiation of treatment on or around the time of menopause [3], [4].

Functional neuroimaging data on the effects of estrogen HT in brain aging and risk for dementia in postmenopausal women are limited. Several lines of evidence suggest that the neuropathological decline in brain function leading to Alzheimer's disease (AD) begins many years before patients develop the full AD clinical syndrome, i.e., disease severe enough to warrant the diagnosis of probable AD (NINCDS-ADRDA diagnostic criteria) [14]. Consistent with a prolonged preclinical disease stage, a number of investigations, including studies from our group, have shown that positron emission tomography (PET) measures of cerebral glucose metabolism vary according to AD genetic risk (apolipoprotein E ε-4 [ApoE-ε4]) and predict cerebral metabolic and cognitive decline in people with mild cognitive complaints (age-associated memory impairment) [15]–[17]. In particular, hypometabolic brain changes in the posterior cingulate, parietal, temporal and eventually prefrontal regions are found with FDG-PET and are associated with cognitive decline and AD.

Other biomarkers of brain function, i.e. structural and functional magnetic resonance imaging and neuropsychological assessments, are also of predictive value for middle-aged and older women at risk for dementia [18]–[37], although studies have varied in the type of HT formulation and their control for pre-study disease state. The present longitudinal findings follow previously reported baseline (cross-sectional) results from this study, in which we reported regional differences in cerebral metabolism in accordance with the type of estrogen component of HT [38] that subjects had been taking at the time of enrollment. The current report summarizes our longitudinal PET findings from a study of postmenopausal women (on 17βE-based or CEE-based HT) who were randomized to continuation or discontinuation of their HT for a two-year observational period. This study utilized functional ([18] fluorodeoxyglucose positron emission tomography) and structural (magnetic resonance imaging [MRI]) brain imaging, along with assessment of cognitive performance to identify biomarkers of preclinical change and propose predictors of cognitive decline. Study inclusion criteria called for postmenopausal women, ages 50–65 years, at risk for dementia, and who had been receiving prescribed HT for at least one year. The main purpose of the overall study was to determine whether HT of any preparation is protective of regional cerebral metabolism and cognitive performance of postmenopausal women with at least one risk factor for AD. Risk factors included having a first-degree relative with AD, known carriership of apolipoprotein E (ApoE-ε4) allele, or personal history of major depression. Brain regions considered appropriate for examination *a priori*, based upon well-established metabolic involvement in the earliest stages of prodromal Alzheimer's disease were the bilateral precuneus/posterior cingulate areas, parietotemporal cortex in the vicinity of the angular and marginal gyri, medial prefrontal cortex given its aging-related metabolic decline, and as described in our baseline analysis, attention was further directed to the medial temporal including the hippocampal area, inferior lateral temporal, and dorsolateral prefrontal cortex, for their roles in cognitive processes vulnerable to early decline in aging individuals [38]. The primary aim focused on regional cerebral metabolism change and the study was statistically powered to detect medium to large effects after appropriate correction for multiple comparisons.

## Methods

The protocol for this trial and supporting CONSORT checklist are available as supporting information; see Checklist S1 and Protocol S1.

Ethics Statement: The study was approved by the Human Research Protection Program at Stanford University, and the Institutional Review Board at the University of California, Los Angeles.

**Figure 1:**
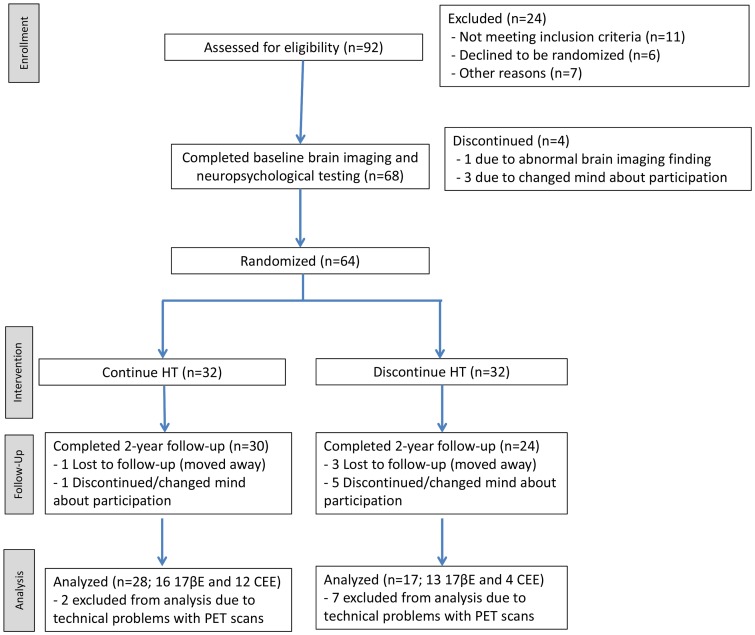
Study Subject Flow.

All subjects were recruited between the dates of January 1, 2004 and October 4, 2007, and 2-year follow-up assessments occurred between April 5, 2006 and October 26, 2009. A flowchart of study subjects is provided in Figure 1. A target sample size of 50 subjects (25 randomized to continue HT and 25 randomized to discontinue HT) completing all procedures at 2-year follow-up was established based on large effect sizes (d∼.9) observed in preliminary data by the senior author [39]. An estimated 70% subject retention rate was initially anticipated, therefore a target of 71 (approximately 35 per randomization group) was established for randomization. However, the observed study retention rate was found to be approximately 80%, and randomization was ended at a target N of 64, 32 subjects per randomization group. At time of enrollment, all subjects met the 2001 Stages of Reproductive Aging Workshop (STRAW) criteria for menopause and were taking continuous (not cyclic) HT. Women in the current analyses included 18 women using 0.625 mg CEE (9 monotherapy, 9 concurrent with progestin) and 36 women using 0.1 mg 17βE (12 monotherapy, 24 concurrent with progestin). All taking concurrent progestin were using Provera, with the exception of 2 subjects taking Prometrium (1 from each HT randomization group). Eligibility screening for the study included willingness to sign consent for all study procedures and undergo randomization to continue or discontinue current HT, psychiatric, physical, and neurological examination, and laboratory blood measures. After screening in, subjects underwent baseline positron emission tomography (PET) and formal neuropsychological testing (complete results to be reported elsewhere), followed by randomization to continue or discontinue HT for a period of two years. For those subjects randomized to stay on HT, the investigators implemented no changes to current HT regimen. During the two-year follow-up period, subjects underwent interim assessments every three months to closely monitor cognition, mood, and serum estradiol levels. If a subject's mood was determined to have declined at interim visits, a referral was made to treating physician for medication management in order to assure mood stabilization and prevent negative effects on brain metabolism and cognition. At the end of two years, subjects repeated all baseline assessments, including PET and neuropsychological testing. Self-reported information from subjects was confirmed by documentation from primary health care providers whenever possible. Duration of endogenous exposure was calculated by subtracting age at menarche from age at menopause. Serum estradiol allowed for monitoring of adherence to randomization.

Inclusion criteria were: 50–65 years of age at time of recruitment, ≥1 year post-complete cessation of menses, ≥1 year current HT use, ≥8 years of education. In addition, all subjects were required to be at elevated risk for dementia, as defined by having a first-degree relative with AD, known carriership of ApoE-ε4 allele, or personal history of major depression [67]–[69]. Exclusion criteria included: history of TIAs, carotid bruits on auscultation, lacunes on MRI, evidence of Parkinson's disease as determined by items 18–31 of the UPDS (39), current depression as determined by a score of ≥8 on the 17-item Hamilton Depression Rating Scale [40], history of drug or alcohol abuse, contraindication for MRI (e.g., metal in body, claustrophobia), history of mental illness other than mood disorders, significant cognitive/functional impairment evidenced by impaired daily function and/or MMSE <24 [41], myocardial infarction within the previous year or unstable cardiac disease, significant cerebrovascular disease evidenced by neurological examination, uncontrolled hypertension (systolic BP >170 mmHg or diastolic BP >100 mmHg), history of significant liver or pulmonary disease, diabetes, cancer, dementia or other condition that could be expected to imminently produce cognitive deterioration, or use of drugs with potential to significantly affect psychometric test results (including centrally active beta-blockers, narcotics, clonidine, anti-Parkinsonian medications, antipsychotics, benzodiazepines, systemic corticosteroids, medications with significant cholinergic or anticholinergic effects, anticonvulsants, warfarin) or phytoestrogen-containing products that could produce estrogenergic agonist and antagonist effects [42], [43].

### Subject Flow, Randomization and Masking

Of 64 subjects who initially underwent randomization, 32 were randomized to continue their existing HT (referred to as HT+) and 32 were randomized to discontinue their HT (HT−). Subsequently, 54 participants completed the two-year follow-up assessment; 30 from the HT+ group and 24 from the HT− group. No significant differences were seen between women who completed or did not complete all study procedures in terms of age, education, IQ, MMSE, years of endogenous estrogen, age at menopause, time since menopause, age at menarche, BMI, presence of the ApoE-ε4 allele, family history of AD, depression history, type of estrogen, use of progestin, or whether they had natural or surgical menopause. Due to technical problems with some of the scans due to movement issues and/or scanner upgrade, two-year follow-up PET data were available for 28 HT+ participants (16 continuing use of 17βE and 12 continuing use of CEE) and 17 HT− participants (13 discontinuing use of 17βE and 4 discontinuing use of CEE). Given the nature of the study design, subjects were aware of their randomization condition (HT+ vs. HT−). Members of the research team who performed evaluations were not aware of the subjects' HT randomization condition, and neuroimaging data was de-identified image to ensure blinded analysis.

Clinical and demographic data analyses were performed using SPSS software version 19.0 (SPSS Inc., Chicago, IL). Two-group t-tests and chi-square tests were used to assess any potential differences in clinical or demographic variables in the two subject groups (HT+ and HT−).

### PET Acquisition and Analysis

Participants fasted 4–6 hours before PET. An intravenous line was placed 10–15 minutes prior to injection of 370 MBq FDG. Uptake of FDG proceeded while subjects were supine with eyes open in a quiet, dimly lit room. Scans were performed 40 minutes post FDG injection using the CTI/Siemens (Siemens Corp, Hoffman Estates, Il) HR+ tomograph (63 image planes).

PET data were analyzed by the statistical parametric mapping method of Friston, Holmes, and colleagues [44]. Briefly, images from all subjects were co-registered and reoriented into a standardized coordinate system using the SPM8 software package courteously provided by the Wellcome Department of Cognitive Neurology, Functional Imaging Laboratory (London). Data were spatially smoothed, and normalized to mean global activity as previously described [45], except that an 8 mm smoothing filter was applied to images prior to statistical analysis. The set of pooled data were then assessed with the t-statistic on a voxel-by-voxel basis, to identify the profile of voxels that significantly differed between subject groups. Brain regions considered appropriate for examination *a priori*, based upon well-established metabolic involvement in the earliest stages of prodromal Alzheimer's disease were the bilateral precuneus/posterior cingulate areas, parietotemporal cortex in the vicinity of the angular and marginal gyri; medial prefrontal cortex given its aging-related metabolic decline; and as described in our baseline analysis, the medial temporal including the hippocampal area, inferior lateral temporal, and dorsolateral prefrontal cortex, for their roles in cognitive processes vulnerable to early decline in aging individuals [38]. To statistically protect against multiple comparisons across the volume of the brain, a Bonferroni-type correction was applied to twelve (left and right) regions specified by a priori hypotheses as described above, and group difference in those regions were noted if p<0.05 after correction; differences in other regions are described here when p<0.0005 before adjustment. SPM analyses were first conducted for group differences in change over time, followed by examination of within-group changes.

## Results

Demographic characteristics of subjects are presented in Table 1 and were reported in full detail in our cross-sectional baseline report [38]. Of 45 women with longitudinal PET data, there were no significant differences between HT randomization groups in first-degree family history of AD (N = 21; 7 HT− women, 14 HT+ women) or history of depression (N = 35 with depression; 13 HT− women, 22 HT+ women; N = 10 without depression; 4 HT− women, 6 HT+ women). There was a significant HT group difference in ApoE-ε4 allele carriers (N = 18; 11 HT− women, 7 HT+ women; χ^2^(1, N = 45) = 6.95, p = .013).

**Table 1:** Study Sample Demographics and Clinical Characteristics (N = 45).

	HT Continuers (n = 28)			HT Discontinuers (n = 17)			
	Mean	SD	Range	Mean	SD	Range	p
Age	58.3	4.5	49,65	57.7	5.6	50,69	0.7 (N.S.)
Years of education	16.0	1.9	12,20	16.6	2.0	12,20	0.3 (N.S.)
Duration of HT use	10.5	4.9	1,20	9.4	6.2	2,21	0.5 (N.S.)
Age at menarche	13.1	1.6	9,16	12.8	1.7	11,16	0.6 (N.S.)
Age at menopause	46.1	7.9	26,59	47.5	4.8	37,54	0.5 (N.S.)
Years of endogenous estrogen exposure	32.7	7.5	13,44	33.9	4.6	23,42	0.5 (N.S.)
BMI	25.7	4.2	19,37	25.8	2.8	22,30	0.9 (N.S.)
Baseline serum estradiol (pg/mL)	36.1	39.9	2,185	43.8	25.0	15,96	0.4 (N.S.)
Baseline FSH (mIU/mL)	58.8	22.8	25,109	62.3	23.9	23,106	0.6 (N.S.)

Women in the follow-up sample who were randomized to discontinue HT did not differ from women who were randomized to continue HT with respect to age, years of education, MMSE scores, age at menopause, surgical or natural menopause, length of HT use, or length of reproductive life. All subjects had MMSE and cognitive performance scores within normal range for cognitively intact persons of corresponding age and educational attainment, at both study entry and two-year follow-up Women initiated HT during perimenopause or within a year following onset of menopause. . More women randomized to continue HT were receiving opposed estrogen (i.e. with progesterone/progestin) than those randomized to discontinue HT. Compliance with randomization was confirmed by assessment of serum estradiol at 3-month interim visits. Estradiol levels were similar between randomization groups at baseline and significantly different at follow-up assessments (e.g. 12-month mean for HT− women = 10.9 pg/mL, HT+ women = 39.5 pg/mL, Z = 4.156, p<.001; 24-month mean for HT− women = 11.3 pg/mL, HT+ women = 32.5 pg/mL, Z = 4.705, p<.001).

### Group Differences in Cerebral Metabolism Change Between Randomization Groups

In direct comparison of two-year regional cerebral metabolic changes occurring between those women randomized to continue HT (n = 28) compared to those randomized to discontinue HT (n = 17), using difference of differences (inter-group) analysis, the medial prefrontal cortex showed significantly greater decline in metabolism in the HT− than HT+ group (t = 4.14, p<.001), due to relative metabolic preservation in women remaining on HT. This was particularly of note since this is a brain region known to metabolically decline with age. Lateral frontal and parietal cortex also were differentially affected between the two randomization groups, with discontinuation of HT being associated with significantly more decline in the right inferior parietal cortex, (t = 5.46, p<.0005) and left frontoparietal area (t = 5.28, p<.0005).

In within-group analysis, women continuing HT exhibited significant increases in the left parietal (t = 6.49, p<.0005) and right frontal areas (t = 6.47, p<.0005), areas which did not meet significance criteria after multiple-comparison correction among women who had discontinued HT, though direct inter-group comparison was also not significant for those areas.

**Figure 2:**
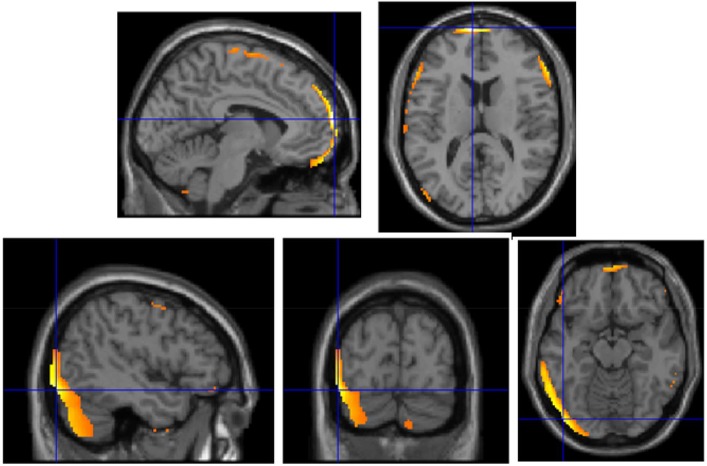
Bilateral medial frontal metabolism (first row) and left temporo-occipital metabolism (second row) were significantly preserved in HT+ women who were ApoE-ε4 negative, compared to HT− women who were ApoE-ε4 negative. Color voxels shown above have significance of p<0.001.

Since a larger proportion of HT− subjects carried the ApoE-ε4 allele than did the HT+ subjects, and the majority of all subjects (27 of 45) were non-carriers of ApoE-ε4, we also examined neuroimaging data among ApoE-ε4 non-carriers only, and inter-group analysis confirmed that the medial prefrontal cortex showed significantly greater decline among those who discontinued HT (t = 6.2, p<0.0005), as did the left temporo-occipital area (t = 5.1, p<0.0005; Figure 2).

### Estrogen Type and Differences in HT Randomization Groups

**Figure 3:**
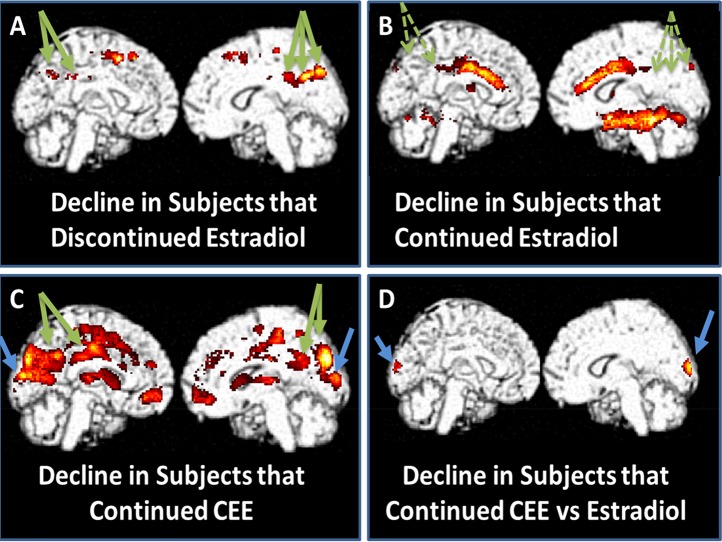
Subjects who discontinued 17β-E demonstrated significant decline in the precuneus/posterior cingulate (green arrows in part A), while this was not seen in subjects that continued 17β-E (green arrows in part B). Subjects who continued CEE underwent significant declines in the primary visual cortex and precuneous/posterior cingulate (blue and green arrows in part C, respectively). In a difference of differences analysis, the primary visual cortex was the region of most significant difference between subjects who continued on CEE versus 17β-E (blue arrow in part D). All color voxels are significant at p<0.005. (Differential effect between women discontinuing estradiol versus those discontinuing CEE was observed in precuneus/posterior cingulate cortex at p<0.01, not shown here.)

For the group of women who discontinued 17βE (n = 13), the most significant region of cerebral metabolic decline within the medial cortical areas occurred in the precuneus/posterior cingulate, the region of the brain that declines most significantly in the earliest stages of Alzheimer's disease (right-sided decline more significant than left: right: t = 4.77, p<0.0005, green arrows, Figure 3a). In contrast, women who continued use of 17βE (n = 16) showed no significant declines in the precuneous/posterior cingulate in either hemisphere (Figure 3b), while women who continued use of CEE (n = 12) demonstrated significant declines in the precuneus/posterior regions bilaterally (left: −4,−20,30, t = 6.48, p<.0005; right: 16,−56,26, t = 4.71, p<.0005).

### Progestin Use and Differences in HT Randomization Groups

**Figure 4:**
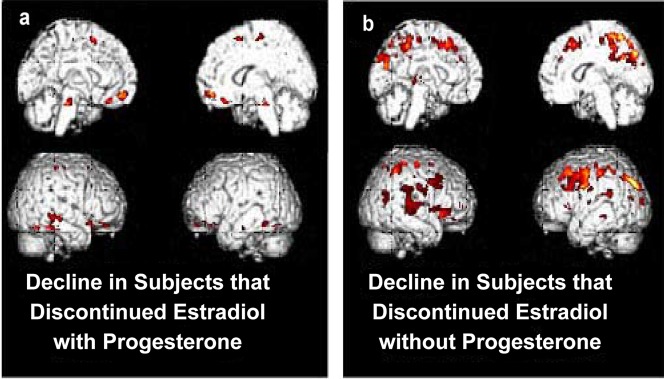
Subjects that discontinued E opposed by progesterone and stayed off HT for two years demonstrated decline in the medial frontal gyrus, while subjects that discontinued unopposed E and stayed off HT for two years demonstrated decline in the precuneous and dorsofrontal cortex. Color voxels shown above have significance p<0.01.

In a direct (inter-group) statistical comparison of two-year change between discontinuation of opposed and unopposed 17βE (n = 6 vs. n = 7), the posterior cingulate was the largest and most significantly different in metabolic change between the two groups (t = 3.95, p = .001, Figure 4b).

**Figure 5:**
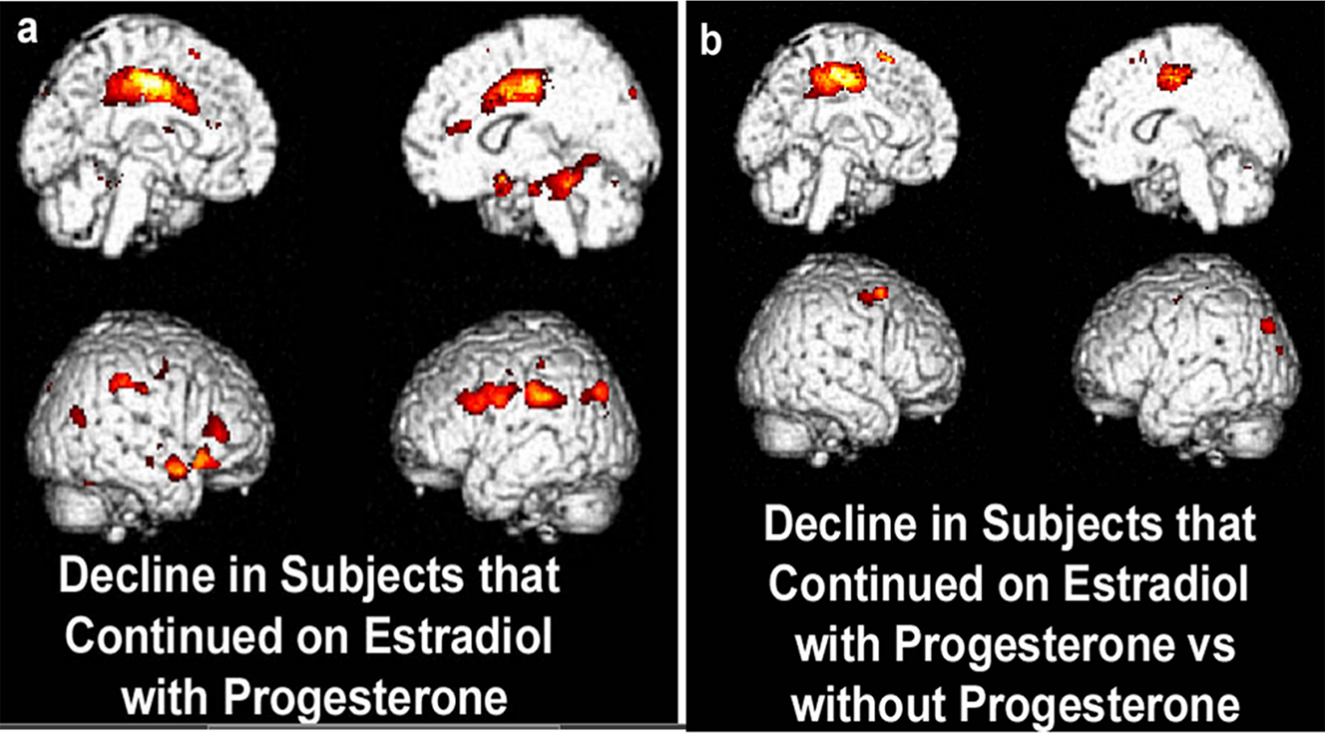
HT+ women continuing on 17β-E with concurrent progesterone demonstrated decreased metabolism with the statistical cluster mapping from the lateral parietotemporal cortex, extending medially through the brain into the posterior cingulate. The posterior cingulate was the most significantly different metabolic area between subjects continuing use of opposed 17β-E versus subjects on unopposed 17β-E. Color voxels shown above have significance of p<0.01 with crosshairs.

In within-group analyses among women continuing use of 17βE concurrent with progestin (n = 12), the largest and most significant area of decline was observed in the left parietotemporal and posterior cingulate cortex (p<.0005, Figure 4a). This region contained 181,370 contiguous voxels at p<.01), (corrected cluster P_FWE,FDR_<0.0005). Among women who discontinued 17βE concurrent with progestin, the most significant area of decline occurred in the medial frontal gyrus (p<.0005, 5a). The precuneus and posterior dorsofrontal cortex were the regions of greatest regional cerebral decline among women who discontinued unopposed estradiol (i.e. without concurrent progestin) (n = 7; p = 0.001, Figure 5b).

Taken together, the two-year longitudinal brain imaging data reflect relative declines in metabolism of the precuneus/posterior cingulate among women who continued opposed 17βE, opposed CEE, and unopposed CEE, suggesting that including progestin in the HT regimen negates the protective effect of continuing 17β-E on posterior cingulate metabolism.

## Discussion

The main findings from this longitudinal study of brain function are as follows:

1. As a group, women who discontinued HT exhibited significant metabolic decline in the medial frontal cortex in comparison to women randomized to continue on HT.
2. Women who discontinued 17βE-based HT, as well as women who continued on CEE-based HT exhibited significant decline in the posterior cingulate/precuneus area, whereas women who continued 17βE-based HT and those discontinuing CEE had no observable metabolic change in this area.
3. Women continuing use of estrogen HT with concurrent progesterone exhibited significant decline in the posterior cingulate/precuneus area regardless of whether the HT was 17βE- or CEE-based.

FDG-PET is a modality used to identify brain abnormalities (parietal hypometabolism and asymmetry) at preclinical stages that predict eventual dementia [46]. Although limited, there is evidence of an association between metabolic activity seen with FDG-PET imaging and increased neurofibrillary tangles, senile plaques, and neuronal loss in patients with verified AD [47]. PET determinations of glucose metabolism in AD show a consistent pattern of reduced glucose use beginning in posterior cingulate, parietal and temporal regions and spreading to the prefrontal cortices [48]. The extent of hypometabolism correlates with severity of cognitive impairment and often shows right/left hemispheric asymmetry at early stages of diseases [49]–[51]. Meguro and associates found that lesions in primate entorhinal cortices, the areas of earliest neuropathological changes in AD, result in hypometabolism in inferior parietal, posterior temporal, and posterior cingulate cortices, all of which are areas of presymptomatic or preclinical hypometabolism observed in PET studies [52].

The current results provide the first prospective randomized direct examination of the effects of continuing versus discontinuing HT upon cerebral metabolism. Similar to our findings at baseline [38], two-year follow-up results revealed differential regional metabolic changes by type of HT formulation. Overall, in a direct statistical comparison between the HT+ and HT- women, the medial prefrontal region showed the largest difference between the two groups, confirming and extending our previously published observation of decreased metabolic activity in CEE users in comparison to 17β-E users at the baseline evaluation of this cohort [38].

For women randomized to discontinue HT, the two-year follow-up imaging data were indicative of differential changes in metabolism according to the type of HT. Women who discontinued 17β-E exhibited metabolic decline in precuneus/posterior cingulate regions, whereas women discontinuing CEE had an increase in metabolic activity in the same region (small N notwithstanding).

The similarity in regional metabolic change between 17β-E users who discontinued HT and CEE users randomized to continue HT, suggests that continued use of CEE is largely ineffective in preserving activity in the precuneus/posterior cingulate region. Unopposed 17β-E appears to provide protection against posterior cingulate decline, and, could potentially play a role in delaying the onset of dementia.

Previous PET studies have demonstrated activational estrogen effects on cerebral function in temporal and parietal regions in both reproductive-aged and healthy postmenopausal women [53]. Healthy women taking HT have shown greater metabolic activity in mesial temporal structures than HT non-users in previous imaging studies, and our results from a group of women at risk for dementia reflect these conclusions. In our pilot analyses of metabolic changes between genders, women using estrogen demonstrated increased metabolism in the lateral temporal cortical regions compared to female non-users and males [54]. These same regions (i.e. lateral temporal and posterior cingulate) exhibit the greatest magnitude of metabolic decline after two years in middle-aged and older persons at genetic risk for AD [55].

A role for 17β-E in the neuroprotection of the precuneus/posterior cingulate area may be inferred from the cumulative data, including previous studies that document the significance of the posterior cingulate as a marker for the advancement of cognitive decline [53], [56]. The first study noting the predictive power of posterior cingulate metabolism for deficit progression in patients with severe memory deficits was performed by Minoshima and colleagues [53]. This observation was followed by several studies that indicated a high predictive power with sensitivity and specificity above 80% for the prediction of rapid progression. Mesial temporal metabolic impairment has been observed as a general feature in patients with memory impairment, including but not limited to patients with MCI and AD [53], [56]–[58].

Our results are consistent with other two-year longitudinal imaging studies of cerebral blood flow which showed greater increases in rCBF in the right hippocampus, bilateral middle temporal gyrus, and left medial frontal gyrus among other regions in HT users in comparison to non-users [59]. When significant differences occurred between groups in patterns of longitudinal change, most of those differences involved HT users showing increased rCBF in a given cerebral region over time, and there were no regions in which women randomized to discontinue HT had greater increases in relative resting rCBF over time [59]. Taken together, these results support the notion that HT is associated with enhanced activity in areas of the brain affected early in the pathological aging.

Comparing our findings of differential effects by the estrogen type (17β-E and CEE), we note a previous study by Smith and colleagues that found that women using HT, regardless of the preparation type, exhibited increased metabolism in the mesial temporal areas compared to non-users [60]. Our results suggest that CEE does not confer the same benefits as 17β-E either in terms of metabolism or cognition. In fact, we previously reported that at baseline women taking 17β-E performed 3 standard deviations higher in verbal memory than women taking CEE, and their verbal memory performance positively correlated with metabolism in Wernicke's area [38], [61].

The present findings provide evidence that the use of 17β-E in postmenopause could be protective against some of the effects of “normal” aging, given the difference in anterior medial prefrontal cortex metabolism observed between women continuing and discontinuing 17β-E. This part of the brain is known to exhibit decreased metabolism as a part of the natural aging process, independent of any neurodegenerative disease [62]. Thus, the increased activity in this region shown by women continuing use of 17β-E is an encouraging result in terms of preserving executive functioning into older age. Both of these metabolic findings confirm the results from our preliminary analyses performed when half of the subjects had completed the protocol [63]. The fact that these findings remained consistent is a testament to their strength and reliability.

Further supporting the observed differences in cerebral metabolism relative to type of estrogen formulation were the effects of progesterone (or lack thereof). For women continuing to use CEE unopposed by progesterone, the largest decrease in metabolism was found in the posterior lateral temporal cortex, extending into the precuneus and middle frontal gyrus. These metabolic declines are consistent with changes in brain areas associated with early stage AD [64].

Preserved metabolism for the posterior cingulate in only those women who continued use of unopposed 17β-E suggests that HT with 17β-E alone may be a viable means to slow the onset of AD symptoms. These findings lead to the conclusion that the strongest neuroprotection of the precuneus/posterior cingulate area is afforded by unopposed 17β-E, and that including progesterone in HT mitigates the benefits conferred by estrogen treatment. To our knowledge, no studies have previously described effects of opposed versus unopposed HT on cerebral metabolism, amplifying the importance of replicating our results for their potentially significant health care implications. The importance of the role of 17β-E in protection of the precuneus/posterior cingulate area is further illustrated by preservation of cognitive abilities in the current study (data not shown) and previous studies that document the significance of the posterior cingulate metabolism as an early marker of cognitive changes consistent with Alzheimer-related dementia [48], [53], [56]–[58].

A number of specific characteristics of this sample deserve mention in order to reasonably summarize the results. There were several considerations that led us to enroll subjects of the 50–65 years age cohort. First, we anticipated that many women 50 years and older would have subjective memory complaints, therefore their risk for developing cognitive decline during the course of the study was increased. Second, changes in regional metabolic patterns precede the actual cognitive decline by many years, even decades [48], so it was important to choose an age group where such changes in the brain may be corroborated by changes in cognitive performance. Another reason for choosing this particular age range is the fact that span of 50 to 65 years of age covers a critical time for cognitive changes before they become assessable by neuropsychological tests. Timing of HT initiation is another important moderator of its effects in the organism. In our study, all but two women had initiated HT at the time of perimenopause or within one year of menopause onset, whereas women in the WHI study initiated HT after age 65 years, years after the onset of menopause. The characteristics of our sample are consistent with the age and timing of HT initiation that is proposed by the window of opportunity theory. Interestingly, our previous findings are consistent with the WHI findings among women receiving CEE with or without progesterone (medroxyprogesterone acetate), wherein women receiving CEE with concurrent progesterone showed declines in delayed verbal memory scores [65].

Prolonged hypoestrogenism (on average 12 years) prior to starting HT in the WHI study may have been the main reason for the negative effects of CEE on indices of brain function. In fact, post-hoc analyses from the WHI study indicate that those women who initiated HT at or around the time of menopause did, in fact, have reduced risk for AD and all-cause dementia [66]. However, the addition of a progesterone component seems to invariably be associated with worse brain function independent of the time at initiation of HT or type of estrogen formulation.

In AD, neurological decline begins in certain brain regions, like the posterior cingulate and lateral temporal, before noticeable symptoms manifest. These regions also tend to show the most metabolic decline in women at risk for dementia, even while they remain outwardly healthy. Deficits of attention and executive function are also seen in persons with mild cognitive impairment as well as with early AD. Therefore, it is essential to determine in further longitudinal studies whether HT-related preservation of metabolism in the critical brain regions can, in fact, postpone this decline, and if so, which type of HT and which populations of women may benefit from using HT.

There are some notable limitations to our study. These results cannot be generalized to healthy postmenopausal women without risk factors for dementia. At the same time, they may be relevant in a number of situations, as some of these risks factors are present in the general population and may be overlooked by researchers in other neuroimaging studies. In addition, the small sample size in our study did not allow for conclusions on cognitive function in the absence of regional metabolic changes. However, this study was aimed at pinpointing metabolic changes as a main outcome variable. Similarly, the small sample precluded assessment of potential interaction effects of genetic risk factors for dementia and use of HT. A separate randomized study of HT on women with and without risk factors for dementia would be able to clarify this issue.

“Healthy user bias” can be applied to our findings since the cohort of women were overall healthy, middle-aged and well educated, so that the observed changes in brain function were within normal ranges for the age category. At the same time it is also a strength of the present investigation that the study sample was unusually homogeneous in terms of demographics, in that all of the present study subjects were women, all were postmenopausal and well-documented with respect to pharmacologic records on hormonal (and other) treatment initiated around the time of menopause, and aged between 50 and 65 years old (e.g., near menopause). In addition, the detailed information obtained by self-report and recorded from the medical records with respect to estrogen use allowed for exploratory subgroup and correlational analyses of cerebral metabolism that have not been feasible in previous investigations assessing longitudinal changes in metabolism of specific brain regions implicated in the dementia.

## Supporting Information

Checklist S1 **CONSORT Checklist.**
(DOC)

Protocol S1 **Trial Protocol.**
(PDF)
