# Supplementary material for: Prospective Randomized Trial to Assess Effects of Continuing Hormone Therapy on Cerebral Function in Postmenopausal Women at Risk for Dementia
Source: PLoS One. 2014 Mar 12;9(3):e89095. doi: 10.1371/journal.pone.0089095 (PMC3951184; doi:10.1371/journal.pone.0089095)
Supplement: Protocol S1 — Trial Protocol. (PDF) [file pone.0089095.s002.pdf]

ESTROGEN USE FOR PROTECTION FROM COGNITIVE DECLINE

Protocol

PROTOCOL

Subject Recruitment

According to the enrollment schedule, subjects recruited during project year 1 will be followed through project year 3; those recruited during year 2 will be followed through year 4; those recruited during year 3 will be followed through year 5. Over the 5-year study period, we will need to retain 50 subjects to achieve the effect size of 0.80. To reach the desired effect size, we will screen by phone approximately 250 subjects (83 subjects per year). We anticipate that 102 subjects will meet preliminary inclusion criteria and will be scheduled for the initial visit (34 subjects per year) and we expect to randomize approximately 71 subjects who meet full inclusion criteria (24 subjects per year), bearing in mind expected rate for the telephone screening (60%), failure to meet inclusion criteria at initial visit (30%) and attrition due to withdrawal from the study either by the PI or by the subject herself (30%). We estimate a 70% rate of subject inclusion in the current proposal based on: a) growing concern on the side of physicians and women alike of the utility of long-term estrogen use in postmenopause; b) interest in definition of the risk for cognitive decline among postmenopausal women and c), brain imaging procedures (in her previous studies, the PI had encountered sustained interest in participation in the studies involving PET imaging to predict possible AD).

Inclusion and Exclusion Criteria

The proposed study design calls for identifying subjects at risk for cognitive decline but not necessarily for developing dementia during a two-year period. Thus, estimates for incipient dementia are not critical for the study design, since incipient dementia is not a primary outcome measure. For the proposed study, we will use the following inclusion criteria:

1. Willingness to sign the Human Subject Protection Consent Form prior to enrollment into the study.
2. Willingness to be randomized to continuation/discontinuation of estrogen replacement therapy.
3. Women 50-65 years of age.
4. One year or more post complete cessation of menses.
5. Current estrogen use for at least one year.
6. Adequate visual and auditory acuity to allow neuropsychological testing.
7. Greater than 8 years of educational achievement to allow adequate neuropsychological testing.
8. Willingness to undergo brain imaging procedures (MRI and PET).
9. Risk for cognitive decline, as defined by one or more of the following:

- Personal history of mood disorder.
- Family history of AD.
- APOE-ε4 allele.

Because cognitive decline may be caused by various conditions, it is critical to exclude subjects with impairment from numerous causes (e.g., vascular disease, etc.). In addition to the above inclusion criteria, we will use the following exclusion criteria:

1. Diagnosis of possible or probable AD [1] or any other dementia (e.g., vascular, Lewy body, frontotemporal).

2. Evidence of neurologic or other physical illness that could produce cognitive deterioration. Volunteers with a history of TIAs, carotid bruits, or lacunes on MRI scan will be excluded. During physical and neurological examination, subjects will be screened for Parkinson disease using the motor examination (items 18-31) of the Unified Parkinson's Disease Rating Scale [2], an instrument with high inter-observer concordance, to quantify presence and degree of symptoms.
3. History of myocardial infarction within the previous year or unstable cardiac disease.
4. Significant cerebrovascular disease, as evidenced by neurological examination, uncontrolled hypertension (systolic BP > 170 or diastolic BP > 100), history of significant liver disease, clinically significant pulmonary disease, diabetes, or cancer.
5. Because medications can affect cognitive functioning, need for a drug that may significantly affect psychometric test results will be reason for exclusion. Use of any of the following drugs will also exclude subjects: centrally active beta-blockers, narcotics, clonidine, anti-Parkinsonian medications, antipsychotics, benzodiazepines, systemic corticosteroids, medications with significant cholinergic or anticholinergic effects, anti-convulsants, warfarin, or sporadic use of phytoestrogen-containing products*.
6. Evidence of depression as determined by a HAMD (Hamilton, 1960) score of 8 or more (17-item version).
7. History of drug or alcohol abuse.
8. Contraindication for MRI scan (e.g., metal in body, claustrophobia).
9. Current episode of mental illness (including mood disorders), or past history of mental illness (excluding mood disorders).
10. Cognitive decline, as evidenced by change in daily functions and/or MMSE <24 (Folstein et al, 1975).

*Because phytoestrogen-containing products can produce estrogen-like agonist and antagonist effects [3, 4], all subjects will be asked not to start use of phytoestrogen-containing products (i.e. soy, tofu, herbs, topical preparations, etc.) during study participation. Similarly, all subjects will be asked not to start use of preparations with putative mood or cognitive enhancing properties (i.e., St. John's Wort, ginko biloba, etc.). We will enroll subjects taking these agents if they have been taking them at a stable dose for at least two months. We will request those women who are already using phytoestrogen-containing products and/or mood/cognition-effecting preparations to continue taking those supplements and to maintain a consistent dosage.

Subject Participation

In the present study, participants will be randomly assigned to continue or discontinue their existing estrogen hormone therapy (HT) for the duration of the 2-year study. The assessment schedule includes: a) screening and identification of subjects who meet inclusion criteria; b) clinical evaluation and blood drawing for general chemistry and other analyses; c) baseline neuroimaging (MRI, PET) and neuropsychological assessments; d) interim assessments; and e) two-year follow-up assessments. All tests performed at baseline will be repeated at a 2-year follow-up. Interim assessments with subjects in between in-person visits will include telephone contact at least every other month. At each three-month interval subjects will be asked to return for a follow-up visit to evaluate their general well-being, overall functioning, and subjective memory performance. Subjects will also be encouraged to contact the research team as needed.

<u>Psychiatric examination</u>. The baseline psychiatric assessment includes a psychosocial history, Structured Clinical Interview for DSM-IV--Patient Edition (SCID), and mental status examination,

and will take approximately hours to complete. Dr. Rasgon will oversee the clinicians that perform the psychiatric assessments and evaluate interrater reliability.

The following standardized rating scales and questionnaire will be used: (1) Structured Clinical Interview for DSM-IV Axis I disorders-Patient Edition (SCID-I/P) [5] is a structured interview that allows for making diagnoses in accordance with DSM IV. The interview takes approximately two hours depending on the pathology of the subject, and can, if necessary, be administered over several sessions. The SCID has screening and skip-out questions that allow for rapid assessment of Axis I diagnoses. The SCID will be administered in its entirety at the initial visit. This will be used to establish presence of any psychiatric diagnoses, current or in remission. (2) Mini-Mental State Examination [6] - a score of 24 or more is needed for a diagnosis of AAMI, 23 or less for AD; (3) Clinical Global Impression Scale (CGI Improvement and Severity Subscales) - used to measure global severity and change from the baseline state of behavior; (4) Hamilton Rating Scale for Depression (17-item version [7]) is an observer rated scale with items assessing symptoms of major depression. The scale has been well validated in the depressed population, and takes a ½ hour to administer. The scale will be administered at the screening visit and every 6 months thereafter. A score above 8 indicates depression, subjects with scores greater than 8 will be excluded from the study.

Physical/neurological examination. Given the numerous physical conditions that may cause cognitive impairment [8], comprehensive physical and neurological examinations will be performed on each patient. The standardized neurological examination yields a quantitative measure of neurological status (developed by Dr. Jeffrey Cummings, Director of the UCLA AD Research Center). In addition, outside medical records are routinely reviewed. We also include a brief handedness questionnaire (see Appendix) and the Unified Parkinson's Disease Rating Scale [2].

Neuropsychological assessment. The neuropsychological test battery includes two components: (1) baseline memory tests; (2) and follow-up measures to assess cognitive decline. The baseline battery includes measures that we expect to be sensitive to subtle changes in cognitive function as well as instruments that appear to have some value in prospectively identifying individuals who eventually decline and develop dementia. These tests are included under the assumption that cognitive changes may emerge in the same functional areas that are affected in the earliest stages of clinically detectable AD. The tests will be given during afternoon sessions to avoid diurnal effects. The neuropsychological test battery will include measures of secondary memory, retrieval from semantic memory/language, visuospatial ability, and frontal functions. The tests were chosen based on prior studies that indicate their ability to predict cognitive decline [9] and sensitivity to predict cognitive change [10]. Tests in the battery will include the Auditory Consonant Trigrams (ACT), Benton Visual Retention Test (BVRT), Boston Naming Test (BNT), Color Trail Making Test (Color Trails 1 & 2), Delis Kaplan Executive Function System (DKEFS) - Color-Word, and Verbal fluency subtests, Rey-Osterrieth Complex Figure Test (RCFT), Wechsler Adult Intelligence Scale-3rd Edition (WAIS-III) - Digit Span, Digit Symbol Coding, and Letter Number Sequencing subtests, and the Wechsler Memory Scale-III (WMS)-Logical Memory I & II subtests. Two subtests from the Wechsler Abbreviated Scale of Intelligence (Vocabulary and Matrix Reasoning) were used to characterize intelligence (IQ).

Laboratory Assessment**.** Laboratory studies are required to identify treatable medical illness that may cause cognitive impairment. We will use the laboratory screening recommended by

consensus groups for the investigation of dementia syndromes [8]: complete blood count with differential, urinalysis, screening test for syphilis, TSH, vitamin B12, drug screen, sodium, potassium, chloride, carbon dioxide, creatinine, glucose, bilirubin, calcium, cholesterol, lactic dehydrogenase, alkaline phosphatase, inorganic phosphorous, total protein, albumin, SGOT/PT, blood urea nitrogen, uric acid, triglyceride, and globulin. If a potentially treatable abnormality is identified, appropriate referral will be made. In addition, estradiol and FSH blood levels will be obtained at the screening visit to verify current estrogen use and menopausal status. Estradiol blood levels will also be obtained every 3 months to monitor compliance of randomization assignment.

PET Acquisition

Participants will fast 4-6 hours before PET. An intravenous line will be placed 10-15 minutes prior to injection of 370 MBq FDG. Uptake of FDG will proceed while subjects are supine with eyes open in a quiet, dimly lit room. Scans will be performed 40 minutes post FDG injection using the CTI/Siemens (Siemens Corp, Hoffman Estates, Il) HR+ tomograph (63 image planes).

Data Analysis

PET data will be analyzed by the statistical parametric mapping method of Friston and colleagues [11] to assess the main hypothesis of differences in HT randomization groups. Briefly, images from all subjects will be co-registered and reoriented into a standardized coordinate system using the SPM8 software package courteously provided by the Wellcome Department of Cognitive Neurology, Functional Imaging Laboratory (London). Data will be spatially smoothed, and normalized to mean global activity as previously described [12], except that an 8mm smoothing filter will be applied to images prior to statistical analysis. The set of pooled data will then be assessed with the t-statistic on a voxel-by-voxel basis, to identify the profile of voxels that significantly differed between subject groups. Brain regions considered appropriate for examination a priori, based upon well-established metabolic involvement in the earliest stages of prodromal Alzheimer's disease, are the bilateral precuneus/posterior cingulate areas, parietotemporal cortex in the vicinity of the angular and marginal gyri; medial prefrontal cortex, given its aging-related metabolic decline; and the medial temporal including the hippocampal area, inferior lateral temporal, and dorsolateral prefrontal cortex, for their roles in cognitive processes vulnerable to early decline in aging individuals [13, 14]. To statistically protect against multiple comparisons across the volume of the brain, a Bonferroni-type correction will be applied to twelve (left and right) regions specified by the a priori hypotheses, and group difference in those regions were noted if $p<0.05$ after correction; differences in other regions will be described here when $p<0.0005$ before adjustment. A number of analyses will be completed after stratifying the subjects according to estrogen HT formulation and various clinical variables, such as presence of APOE-4.

Clinical, demographic, and neuropsychological data will be analyzed using SPSS software version 13.0 (SPSS Inc., Chicago, IL). Two-group t-tests and chi-square tests will be used to assess any potential differences in clinical or demographic variables in the two subject groups (HT continuation or HT discontinuation). Paired t-tests will be used to assess changes in neuropsychological test performance between HT randomization groups to allow for consideration of correspondence with regional cerebral metabolism changes. Significance for

these analyses will be set at $p<.05$ level without Bonferonni correction, given the small sample sizes and exploratory nature of these analyses. Further analyses will be conducted to assess differences according to estrogen HT formulation.

Risks to the Subjects

Human Subjects Involvement and Characteristics. This project will screen approximately 250 postmenopausal female subjects, 50-65, in relatively good physical health who either, at risk for cognitive decline (personal or family history of mood disorder, hypothyroidism, family history of AD, APOE-ε4 allele). Exclusion criteria include: a) diagnosis of possible or probable AD, or any other dementia (e.g., vascular, Lewy body, frontotemporal), b) evidence of neurologic or other physical illness that could produce cognitive deterioration. Volunteers with a history of TIAs, carotid bruits, or lacunes on MRI scan will be excluded. During physical and neurological examination, subjects will be screened for Parkinson disease using the motor examination (items 18-31) of the Unified Parkinson's Disease Rating Scale, an instrument with high inter-observer concordance, to quantify presence and degree of symptoms, c) history of myocardial infarction within the previous year or unstable cardiac disease, d) significant cerebrovascular disease, as evidenced by neurological examination, uncontrolled hypertension (systolic BP > 170 or diastolic BP > 100), history of significant liver disease, clinically significant pulmonary disease, diabetes, or cancer, e) use of any of the following drugs will also exclude subjects: centrally active beta-blockers, narcotics, clonidine, anti-Parkinsonian medications, antipsychotics, benzodiazepines, systemic corticosteroids, medications with significant cholinergic or anticholinergic effects, anti-convulsants, warfarin, or sporadic use of phytoestrogen-containing products, f) evidence of depression as determined by a HAMD score of 8 or more (17-item version), g) history of drug or alcohol abuse, h) contraindication for MRI scan (e.g., metal in body, claustrophobia), i) current episode of mental illness (including mood disorders), or past history of mental illness (excluding mood disorders), j) cognitive decline, as evidenced by change in daily functions and/or MMSE <24.

Subject involvement will consist of: a) screening and identification of subjects who meet inclusion criteria; b) clinical evaluation (structured psychiatric interview, physical/neurological examination) and blood drawing for general chemistry and other analyses; c) baseline neuroimaging (MRI, PET) and neuropsychological assessments; and d) follow-up assessments over 2 years. Contact with subjects in-between visits will include telephone contact at least every other month. At each three-month interval subjects will be asked to return for a follow-up visit to evaluate their general well-being, overall functioning, and subjective memory performance. Subjects will also be encouraged to contact the research team as needed.

Sources of research material. Existing medical records will be used to determine eligibility of subject for study entry. In addition, the following materials will be gathered specifically for research purposes: blood samples for screening purposes and to monitor compliance, subjects' responses to rating scales and questionnaires, and PET and MRI scan data. Most subject data will come from four primary sources. First, face to face semi-structured interviews that will cover several aspects of medical, psychiatric and cognitive functioning. These instruments are described in the Research Design and Methods section of the proposal. These instruments have previously been used and validated and present no foreseeable risk to the subjects other than the inconvenience of the time it takes to administer them. The second primary source of

data is physical/neurological examination, as described in the Research Design and Methods. The third primary sources of data are laboratory assessments of blood. Finally, the fourth primary source of data is neuroimaging (PET, MRI). While human subjects will be studied at Stanford University, the key that links the unique subject number to subject identifiers will be maintained at Stanford only (where the P.I., Dr. Rasgon, is located). Each subject studied at Stanford will be assigned a unique subject number. This number will not contain the subjects name, initials, date of birth, hospital ID number, or any other information that might be used to directly or indirectly identify the subjects. Dr. Silverman at the UCLA site will not hold or have access to the key identifying the study subjects, nor will he directly interact with the study subjects; he will be involved strictly at the level of data analysis, as described above.

Potential Risks. The procedures involve minimal potential risks. Blood drawing, though a routine part of general medical examinations, entails a small possibility of such discomforts as bleeding, bruising, lightheadedness, fainting, or rarely, infection. Neuropsychological testing may induce feelings of failure, frustration, or anxiety. However, our psychometrist is experienced in assessing persons with memory impairment and conveys a relaxed and confident attitude. A minority of subjects experience anxiety and claustrophobia during MRI scanning. Potential drug (e.g. estrogen) side effects are monitored closely; medications are discontinued as indicated medically and appropriate interventions provided. The PET scan exposes subject to a small amount of radiation. The radiation dose the subjects receive is well within the accepted limits of the Nuclear Regulatory Commission for radiation workers.

Adequacy of Protection Against Risks

Recruitment and Informed Consent. Currently, the PI is working as an Associate Professor and as the Associate Director of the Women's Wellness Program in the Department of Psychiatry and Behavioral Sciences at the Stanford University School of Medicine. The PI has had a longstanding interest in the interaction between reproductive hormones and brain function, and she was a physician in the WHIMS study at UCLA. In her new capacity, the PI will have access to the resources of the Women's Wellness Clinic, and there is strong enthusiasm from all collaborators at Stanford to work on this project.

One of the PI's primary responsibilities within the University is collaboration with the Center for Research on Women's Health and Reproductive Medicine at Stanford. The Center, with Dr. Giudice as Director, provides the infrastructure for basic, clinical, and translational research grants and contracts in women's health. In addition, Dr. Guidice and Dr. Stephanic (the PI for WHI study here at Stanford) will be at the core of the recruitment process. This collaboration will allow for the recruitment of subjects from the PREMPRO arm of the WHI study based here at Stanford University.

The main source of recruitment will be Stanford University Gynecology (Gyn) Division and Clinic. Clinic is on the third floor of the Stanford University Clinics in the Boswell Building, occupying 4,000 sq. ft. Immediately adjacent to the Clinic is the Center for Research on Women's Health and Reproductive Medicine. In the Center are the Clinical Coordinators and support staff for all clinical studies in the Center. There are two conference rooms in the Clinic where patients may be screened, protocols reviewed, and informed consent obtained. The GYN Clinic sees approximately 1,000 new patients per month. More than 45% of them are between

the ages of 45 and 70. Of these women, approximately 52% of perimenopausal women are currently taking estrogen, and 22% of postmenopausal women are taking estrogen. About 47% of the women taking estrogen are deciding to discontinue. Additionally, several local primary care clinics refer women for potential inclusion into research studies. Additional resources include: 39 families of AD probands from the Alzheimer Disease Family Study; a research team that has continued rapport with these families and is experienced in performing the proposed examinations; the multi-campus geriatric psychiatry and neurology group;Stanford University, Alzheimer's Disease Center and Stanford University VAMC. If a woman shows interest in the study, the study clinician and/or one of the investigators will meet with the woman to describe the study. All interviewing and testing procedures will be explained to the potential subject in detail. The research assistant will document consent by asking the subject a series of questions to verify comprehension of study procedures, risks and benefits. Consent will then be obtained.

Protection Against Risk. Subjects will be assured that they do not need to answer any question on the psychiatric, or cognitive function scales that they do not wish to address. Concerning the venipuncture, the minimum amount of blood necessary for analysis will be obtained.

Potential Benefits of the Proposed Research to the Subjects and Others. Potential benefits for subjects include the complete diagnostic assessment, which may identify underlying medical disorders so that appropriate referrals for treatment can be made. Subjects often express great satisfaction from knowing they have been able to make a personal contribution to the advancement of scientific knowledge and to the search for solutions to health problems – either their own or others'. For society, the increased understanding gained through this study may provide significant leads for treatment and prevention of AD, thus benefiting many older persons. The benefits, then, clearly outweigh the risks.

Because the procedures are time-consuming and subjects do not necessarily receive direct clinical benefits from them, subjects will receive a $25 honorarium per visit and $25/hour (total of $100) for each MRI/PET scan, which also helps cover their travel costs.

The proposed research will help to define guidelines for estrogen use among postmenopausal women. Specifically, it will provide information regarding the effects of discontinuation from estrogen use on cognitive functioning.

Importance of the Knowledge to be Gained. There is little risk to participating in a study assessing the effects of continuation/discontinuation of estrogen use. Yet there is much to gain from identifying changes in brain metabolism and cognitive function, which may result from discontinuation of estrogen use.
a) Individual: Subjects will receive a detailed psychiatric, physical, and neurological examination, including laboratory and cognitive assessment, and neuroimaging.
b) Societal: Results of the study may further understanding of the possible protective effects of estrogen from cognitive decline.

Gender and Minority Inclusion

Inclusion of Women and Minorities. Women are the subjects under investigation and there are no inclusion/exclusion criteria related to minority status. The study population will in part be

drawn from women's health clinics at Stanford and the surrounding community.

Inclusion of Children. As the research questions investigated in this project are specific to postmenopausal women, children will be excluded from the study.

Other Human Subjects Considerations. The characteristics of the proposed study populations are described in the inclusion and exclusion, evaluation, and research procedure portions of the Research Design and Methods section. The PI will routinely spend time to address any questions about the study and provide counseling and general information about cognitive decline and related issues.

Subjects are informed that information on individual genotypes is not provided to them because investigators are blinded to individual results and the analyses are not performed in a licensed clinical laboratory. Subjects who wish information on their genotypes are discouraged from obtaining such results if they do not have dementia, since APOE is not considered a predictive test and the potential psychological consequences of such information could be problematic [15]. Subjects who insist on such information are referred to the Clinical Genetics Division at Stanford University for counseling and further testing from a licensed clinical laboratory as indicated.

Only a small proportion of subjects is expected to develop cognitive decline during the study period. Thus, one concern is that some subjects are being exposed to potential side effects of randomization (in case of continued use, breast cancer risk; for those who discontinue, osteoporosis, etc.) for a condition that they will not develop. However, of the subjects who do not develop cognitive decline during the two-year follow-up period, a significant proportion is at risk for it and eventual AD in future years. Estrogen use if shown to be neuroprotective, may have beneficial effects for them as suggested by epidemiological studies that show estrogen use decreases risk for development of AD dementing process and brain metabolic alterations which begin years prior to clinical confirmation of the disease.

The responsibility for medical, psychiatric, and psychological care of subjects will remain with the subject's primary physician. The PI checks results of laboratory tests soon after they are received. Should abnormal findings be discovered at any time during participation in the project, the participant, and the responsible physician and, if appropriate, responsible relative is informed. Should the participant not have a physician, a referral is made to one in the patient's geographic area. With the subject's consent, copies of laboratory results are sent to their physician.

In conjunction with the Stanford University Human Subjects Protection Committee, all legal and ethical safeguards for participants are implemented. All participants receive a copy of the Subject's Bill of Rights prior to giving consent to participate. The informed consent form is read with each participant, allowing time for questions following each paragraph. Thus, the procedures, potential risks and benefits, right to withdraw and confidentiality are presented in a thorough and uniform manner to all participants.

Data and Safety Monitoring. Adverse events will be evaluated at each study visit and reported regardless of randomization outcome. Any event not previously documented in the study will be

recorded, including the nature of the individual experience, date and onset time, duration, severity, and potential relationship to intervention. Any changes in general well-being, corrective treatment or prescription of concomitant medication/s also will be recorded. Previously recorded adverse events will be reviewed at each visit. We will use nonspecific solicitation of adverse events by asking "Do you feel different in any way since starting the discontinuation of estrogen use?"

Adverse event severity will be rated as mild (noticed discomfort but no disruption of daily activity), moderate (discomfort affects daily activity), or severe (inability to perform daily activity). All adverse events will be followed, and any serious adverse event (e.g., life threatening, incapacitating) will be reported to the health authorities, including NIH, local IRB, and the GCRC Office of Research Participant Advocacy (ORPA). An external advisory group comprised from the national experts in the field of reproductive neuroendocrinology and neuroimaging (Drs. Rubinow and Drevets) will review cognitive and brain imaging data blinded to the intervention every 6 months. Subjects in either arm who will exhibit worsened performance on cognitive tests will have an immediate work-up done, including neuroimaging (PET/MRI) done to evaluate potential changes in brain function and will be removed from the study.

References

1. McKhann, G., Drachman, D., Folstein, M., Katzman, R., Price, D., and Stadlan, E. (1984) Clinical diagnosis of Alzheimer's disease: report of the NINCDS-ADRDA Work Group under the auspices of department of health and human services task force on Alzheimer's disease. Neurology. **34**, 939-44.
2. Fahn, S., Elton, R., and Committee, M.o.t.U.D., *Unified Parkinson's disease rating scale*, in *Recent Development in Parkinson's Disease*, S. Fahn, et al., Editors. 1987, Macmillian: New York. p. 153-163.
3. Polkowski, K. and Mazurek, A. (2000) Biological properties of genistein. A review of in vitro and vivo data. Acta Pol Pharm. **57**, 135-55.
4. Vincent, A. and Fitzpatrick, L. (2000) Soy isoflavones: are they useful in menopause? Mayo Clin Proceed. **75**, 1174-84.
5. Spitzer, R.L., Williams, J.B., Gibbon, M., and First, M.B., *Structured clinical interview for DSM-IV*. 1996, New York: NYU Psychiatric Institute.
6. Folstein, M., Folstein, S., and McHugh, P. (1975) "Mini-mental state": a practical method for grading the cognitive state of patients for the clinician. J Psychiatr Res. **12**, 189-98.
7. Hamilton, M. (1980) A rating scale for depression. Journal of Neurology, Neurosurgery, and Psychiatry. **23**, 49-65.
8. Small, G., La Rue, A., Komo, S., Kaplan, A., and Mandelkern, M. (1997) Mnemonics usage and cognitive decline in age-associated memory impairment. Int Psychogeriatr. **9**, 47-56.
9. Hänninen, T., Hallikainen, M., Koivisto, K., Helkala, E., Reinikainen, K., Soininen, H., Mykkänen, L., Laakso, M., and Pyörälä, R., PJ. (1995) A follow-up study of age-associated memory impairment: neuropsychological predictors of dementia. J Am Geriatr Soc. **43**, 1007-15.
10. Small, G., La Rue, A., Komo, S., Kaplan, A., and Mandelkern, M. (1995) Predictors of cognitive change in middle-aged and older adults with memory loss. Am J Psychiatry. **152**, 1757-64.
11. Friston, K., Holmes, A., Worsley, K., Poline, J., Frith, C., and Frackowiak, R. (1995) Statistical parametric maps in functional imaging: a general linear approach. Hum Brain Mapp. **2**, 189-210.

12. Silverman, D.H., Munakata, J.A., Ennes, H., Mandelkern, M.A., Hoh, C.K., and Mayer, E.A. (1997) Regional cerebral activity in normal and pathological perception of visceral pain. Gastroenterology. **112**, 64-72.
13. Small, G., Mazziotta, J., Collins, M., Baxter, L., Phelps, M., Mandelkern, M., Kaplan, A., La Rue, A., Adamson, C., and Chang, L. (1995) Apolipoprotein E type 4 allele and cerebral glucose metabolism in relatives at risk for familial Alzheimer disease. JAMA. **273**, 942-7.
14. Reiman, E., Caselli, R., Yun, L., Chen, K., Bandy, D., Minoshima, S., Thibodeau, S., and Osborne, D. (1996) Preclinical evidence of Alzheimer's disease in persons homozygous for the epsilon 4 allele for apolipoprotein E. N Engl J Med. **334**, 752-8.
15. Relkin, N., Kwon, Y., Tsai, J., and Gandy, S. (1996) The National Institute on Aging/Alzheimer's Association recommendations on the application of apolipoprotein E genotyping to Alzheimer's disease. Ann N Y Acad Sci. **802**, 149-176.
